# Pyrolysis Atmospheres and Temperatures Co-Mediated Spectral Variations of Biochar-Derived Dissolved Organic Carbon: Quantitative Prediction and Self-Organizing Maps Analysis

**DOI:** 10.3390/molecules28052247

**Published:** 2023-02-28

**Authors:** Huiying Zhang, Jinzhi Ni, Wei Qian, Shuhan Yu, Yu Xiang, Liuming Yang, Weifeng Chen

**Affiliations:** Key Laboratory for Humid Subtropical Eco-Geographical Processes of the Ministry of Education, Fujian Provincial Key Laboratory for Plant Eco-Physiology, School of Geographical Sciences, Fujian Normal University, Fuzhou 350007, China

**Keywords:** pyrolysis atmospheres, biochar-derived dissolved organic carbon, spectral characteristics, quantitative analysis, self-organizing maps

## Abstract

Biochar-derived dissolved organic carbon (BDOC), as a highly activated carbonaceous fraction of biochar, significantly affects the environmental effect of biochar. This study systematically investigated the differences in the properties of BDOC produced at 300–750 °C in three atmosphere types (including N_2_ and CO_2_ flows and air limitation) as well as their quantitative relationship with biochar properties. The results showed that BDOC in biochar pyrolyzed in air limitation (0.19–2.88 mg/g) was more than that pyrolyzed in N_2_ (0.06–1.63 mg/g) and CO_2_ flows (0.07–1.74 mg/g) at 450–750 °C. The aliphaticity, humification, molecular weight, and polarity of BDOC strongly depended on the atmosphere types as well as the pyrolysis temperatures. BDOC produced in air limitation contained more humic-like substances (0.65–0.89) and less fulvic-like substances (0.11–0.35) than that produced in N_2_ and CO_2_ flows. The multiple linear regression of the exponential form of biochar properties (H and O contents, H/C and (O+N)/C) could be used to quantitatively predict the bulk content and organic component contents of BDOC. Additionally, self-organizing maps could effectively visualize the categories of fluorescence intensity and components of BDOC from different pyrolysis atmospheres and temperatures. This study highlights that pyrolysis atmosphere types are a crucial factor controlling the BDOC properties, and some characteristics of BDOC can be quantitatively evaluated based on the properties of biochar.

## 1. Introduction

Biochar has been increasingly applied as a carbonaceous material for environmental restoration, soil quality improvement, carbon emission reduction, and mitigation of climate change [1,2]. Once biochar enters into the environment, it will be weathered, oxidized, microbe-decomposed, and bound to release biochar-derived dissolved organic carbon (BDOC) [1,3]. Compared with biochar and natural dissolved organic carbon (DOC) in the environment, BDOC presents some unique properties and induces positive and negative impacts on the eco-environment [4,5]. For example, BDOC can provide many available carbons for microbial growth [6]. BDOC can promote the transport or immobilization of pollutants as well as facilitate the photochemical transformation of organic pollutants (diethyl phthalate, methylene blue), thus enhancing or reducing the hazard of pollutants in the environment [7,8,9]. BDOC contains aromatic compounds with N atoms, phenols, heterocyclic N compounds, and PAHs, which have high biotoxicity [10]. Considering that the environmental effects of biochar are closely related to BDOC, it is highly necessary to explore the compositions and physicochemical properties of BDOC.

The feedstock types and pyrolysis temperature as two key factors controlled the properties and compositions of BDOC [11,12]. For example, the BDOC content in manure biochar is higher in comparison with plant biochar [13]. Additionally, BDOC content in the biochar from cellulose- and hemicellulose-rich materials (e.g., grass) is higher than that from lignin-rich materials (e.g., wood) [14]. The characteristics of BDOC are also pyrolysis-temperature-dependent. The higher pyrolysis temperature supports a lower release amount of BDOC from biochar [15], which is dominated by low molecular weight components [16]. Meanwhile, high pyrolysis temperature decreases the proportion of fulvic-like and aliphatic substances in BDOC but increases the proportion of humic-like highly aromatic substances [17]. Moreover, the pyrolysis atmosphere types are also an important factor controlling the properties of biochar. The publication reported that the properties of biochar (elemental composition, ash content, volatile matter content, specific surface area, oxygen-containing functional groups) were closely related to the pyrolysis atmosphere types (e.g., N_2_ and CO_2_ flows, and air limitation) [18]. The characteristics of BDOC are closely related to these properties of biochar (e.g., elemental composition, volatile matter content, oxygen-containing functional groups) [19], whereas the properties of BDOC produced in different pyrolysis atmospheres remain unknown. Moreover, though some publications have demonstrated that the properties of biochar controlled the characteristics of BDOC, the quantitative relationships between the characteristics (e.g., releasable content and content of different organic components) of BDOC and the properties of biochar are not fully clarified. Establishing the quantitative relationships between the characteristics of BDOC and the properties of biochar is helpful for predicting the characteristics of BDOC based on the properties of biochar.

Presently, ultraviolet-visible (UV-vis) and three-dimensional excitation–emission matrix (3D-EEM) fluorescence spectrums are frequently applied to explore the properties (including humification, aromaticity, molecular weight, and polarity) of BDOC [20,21]. Meanwhile, the EEM-PARAFAC (parallel factor) method was frequently used to quantify the main components (including humic-, fulvic-, and protein-like substances) of BDOC [15,22]. Furthermore, an advanced stoichiometric algorithm, self-organizing maps (SOM), can map complex 3D-EEM to two-dimensional space with a high visualization degree through nonlinear transformation and retain all its topological and metric properties [20,23]. More interestingly, SOM, in combination with EEM and PARAFAC, gives a clear image that effectively classifies DOC samples according to their fluorescence characteristics [20]. Therefore, combining SOM with EEM and PARAFAC to analyze the spectral characteristics of BDOC is helpful to deeply excavate the fluorescence properties and organic components of BDOC changing with the pyrolysis temperatures and atmospheres.

In this study, BDOC was extracted from the biochars prepared in three atmospheres (N_2_ flow, CO_2_ flow, and air limitation) at 300~750 °C. Firstly, the co-influences of pyrolysis atmospheres and temperatures on the characteristics of BDOC (e.g., DOC content, aromaticity, molecular weight, polarity) were analyzed. Secondly, the quantitative relationships between the characteristics (e.g., releasable content and content of different organic components) of BDOC and the properties of biochar were clarified. Lastly, EEM-SOM and PARAFAC-SOM were used to visualize the difference and classify the BDOC from different pyrolysis atmospheres and temperatures based on the fluorescence properties and organic components of BDOC. This work will be helpful for deeply systematically understanding the properties and potential environmental effects of the BDOC from different pyrolysis conditions as well as quantitatively predicting the characteristics of BDOC according to the properties of biochar.

## 2. Materials and Methods

### 2.1. Biochar Preparation and BDOC Extraction

Pine sawdust and wheat straw as two different raw biomasses were used in this study, and they were collected from a mill in Jiangxi province and a farmland in Henan province, China, respectively. The main organic components of different biomasses are shown in Table S1 (from Supplementary Materials). Biochar was prepared at four pyrolysis temperatures (300 °C, 450 °C, 600 °C, and 750 °C) in three pyrolysis atmosphere types (including N_2_ and CO_2_ flows and air limitation). Before pyrolysis, the two feedstocks were washed with ultrapure water and dried at 80 °C. Additionally, the biomasses were cut or milled into the fragment with length less than 1 cm. Next, they were placed in a quartz tube, then pyrolyzed using an electronic furnace (O KTF1200) for 5 h. For pyrolysis taking place in N_2_ flow atmosphere or CO_2_ flow atmosphere, the pyrolysis process was carried out with a gas flow rate of 0.5 L/min and a ramp rate of 20 °C/min. For pyrolysis taking place in air limitation, approximately 8.0 L of air remained in the quartz tube of the furnace, and the operation was performed without gas purging to simulate the incomplete combustion of biomass. Other operating procedures were consistent with the pyrolysis process in N_2_ flow and CO_2_ flow. Finally, the pyrolysis product (biochar) was removed after cooling to lower than 50 °C, ground, and passed through a sieve of 100-mesh, and then stored in a dryer. The volatile matter content (weight percentage, wt.%) was measured by heating biochar samples at 450 °C for 1 h, and the ash content (wt.%) was measured by heating biochar samples at 750 °C for 4 h in a muffle oven [18]. Fixed C content (wt.%) equals the mass of biochar sample minus the total weight percentages of ash and volatile matter. The C, N, and H contents (wt.%) were determined by an elemental analyzer. The content (wt.%) of O in biochar equals the mass of biochar sample minus the total contents of H, C, N, and ash [18]. Triplicates were performed for each sample.

Biochar (0.5 g) and 0.01 mol/L NaCl solution (40 mL) were transferred into a brown glass vial, and the vial was placed in a thermostatic oscillation chamber (25 °C, 200 r/min) and shaken for 7 days. After 7 days, the vial was taken out and stood for 24 h, then the supernatant was filtered through a 0.45 μm membrane. Finally, the filtrate was stored at 4 °C for further characterization. A total organic carbon analyzer (TOC-V-CPH, Shimadzu, Japan) was used to quantify the BDOC content. Three replicates were set for each sample.

### 2.2. UV-vis Analysis

Before UV-vis spectrum scanning, the DOC content of all BDOC samples was diluted to less than 10 mg/L to eliminate the effect of internal filtration. The absorbance value of BDOC was measured by a UV-vis spectrophotometer (Shimadzu). Ultrapure water was used as the background value. The scanning wavelength was 190–800 nm, the optical path distance was 1 cm (the thickness of the colorimeter was 1 cm), and the wavelength step was 1 nm. The characteristics of BDOC were indicated by UV-vis indexes (including absorption coefficient a_m_ (m^−1^), SUVA_254_ (L/(mg-C·m)), A_220_/A_254_, and S_275∓295_). The absorption coefficient (Figure S1 from Supplementary Materials) a_m_ (m^−1^) was obtained from the following equation [24]: a_m_ =2.303 A/L. Wherein A is the absorbance, and l is the path length of the optical cell in meters and equals 0.01 m here. Herein, SUVA_254_ = a_254_/C_DOC_ [25], C_DOC_ (mg/L) was the DOC concentration of BDOC solution; A_220_/A_254_ was the ratio of absorbance at wavelength 220 nm to 254 nm [19]. S_275–295_, a spectral slope, was obtained using linear regression to fit the exponential transformed absorption coefficients in the wavelength intervals of 275–295 nm [26].

### 2.3. Fluorescence Spectrum and EEM-PARAFAC Analysis

A fluorescence device (F-7100, Hitachi) was used to scan the 3D-EEM spectrum of BDOC. Before spectral scanning, the DOC concentration of BDOC was diluted to less than 10 mg/L in order to eliminate the effect of internal filtration [7]. The excitation (Ex) wavelength range of the EEM was set to 200–550 nm (scanning interval was 5 nm), and the emission (Em) wavelength range was set to 220–600 nm (scanning interval was 2 nm). The blank and Raman correction of ultrapure water was performed for every 10 samples. MATLAB R2020a software was used to run the DOMFluor Toolbox, and PARAFAC modeling analysis was performed on the 3D fluorescence spectrum [27]. The specific modeling methods were given in Text S1 and our previous publication of Zhang et al. [28]. Finally, in this study, four organic components (C1, C2, C3, C4) were extracted from 72 BDOC samples. Additionally, the humification index (HIX) can be obtained from the fluorescence spectrum of BDOC, and the higher HIX value indicates a higher humification degree of BDOC [29]. HIX was calculated as the area of Em intensity of 435–480 nm divided by the area of Em intensity of 300–345 nm, at an excitation wavelength of 254 nm.

### 2.4. EEM-SOM and PARAFAC-SOM Analysis

The detailed algorithm of SOM has been described by Kohonen [30]. In detail, as an artificial neural network method, SOM uses the unsupervised competitive learning mechanism. Through multiple iterations, SOM makes the map vector more approximate to the sample vector. Meanwhile, the distance between the sample vector and the map vector is judged by Euclidean distance. Once the optimal neighborhood map vector is determined, the weights of the map vector and its neighboring neurons will be regulated and gradually transferred to the sample vector [23]. Herein, the construction of the SOM model was based on the SOM toolbox (version 2.0) in Matlab R2021a [31]. The 3D-EEMs data needed to transform into 2D-EEMs data before SOM training. Based on EEM and PARAFAC of all BDOC, two SOM models were used: (1) EEM-SOM model: a 72 (sample numbers) × 6351 (191 × 71 Ex-Em pairs) matrix; (2) PARAFAC-SOM model: a 72 (sample numbers) × 4 (the abundances of four extracted fluorescence components) matrix. The input data need to be normalized (variance 1, mean 0) and run with default settings.

### 2.5. Statistical Analysis 

Independent sample T-test and probability (*p*) less than 0.05 were used to determine the significance of difference. Quantitative relationships between the characteristics of BDOC and the stoichiometry of biochar were established by linear regression, exponential regression, and multiple linear regression using SPSS 25.0 and Origin 2021b.

## 3. Results and Discussion

### 3.1. Elemental Compositions and Properties of Biochar

The C, N, O, and H contents of biochar were presented in Table 1. For biochar pyrolyzed in CO_2_ flow, its C content initially increased and then decreased with a persistently increased temperature. For biochar produced in N_2_ flow, its C content continuously increased as the temperature was persistently increased. Very differently, for biochar pyrolyzed in air limitation, its C content reduced as the temperature was persistently increased. Meanwhile, the C content of biochar pyrolyzed in N_2_ and CO_2_ flows was significantly higher compared with that pyrolyzed in air-limitation pyrolysis at each temperature. This is ascribed to air limitation containing residual O_2_ enhancing the oxidization and decomposition of organic matter in biomass [18]. Meanwhile, the CO_2_ facilitated the transformation of biomass into CO and volatile compounds at the higher temperature of 750 °C [32] (Table 1), whereas N_2_ as an inlet atmosphere could not react with biomass. For each pyrolysis atmosphere, the increased temperature decreased the contents of H and O in biochar (Table 1). Pine sawdust biochars pyrolyzed in CO_2_ and N_2_ flows exhibited an approximate H content higher than biochar pyrolyzed in air limitation. Differently, wheat straw biochar pyrolyzed in air limitation and CO_2_ flow exhibited an approximate H content lower than biochar pyrolyzed in N_2_ flow. The difference between the biochars from the two different biomasses was ascribed to their different organic compositions. Wheat straw is a hemicellulose-rich biomass (Table S1 from Supplementary Materials), and hemicellulose in biomass preferred to be decomposed in CO_2_ flow and air limitation compared with N_2_ flow, and H was emitted in the form of the volatile substances and gases (e.g., aliphatics) during the decomposition of hemicellulose [18,33]. Pine sawdust biochar pyrolyzed in air limitation contained more O compared with biochar pyrolyzed in CO_2_ and N_2_ flows, which was ascribed to air limitation being preferred to oxidize the biomass into volatiles (e.g., phenols) compared to CO_2_ and N_2_ flow [34]. 

The volatile matter contents of biochars were shown in Table 1. For each pyrolysis atmosphere, high pyrolysis temperature supported the gasification of volatile matters, thus declining the volatile matter content in biochars. At each pyrolysis temperature, the volatile matter contents of biochar from the three pyrolysis atmospheres were equivalent, excluding wheat straw biochar of 750 °C with the sequence of CO_2_ flow > N_2_ flow > air limitation. This is because hemicellulose in wheat straw is richer than that in pine sawdust (Table S1 from Supplementary Materials). At the higher temperature of 750 °C, CO_2_ could react with hemicellulose and facilitated its decomposition into volatile matters (C_6_~C_20_ aliphatic substances) retained in biochar, whereas air limitation enhanced the oxidization of hemicellulose into gases (including CO_2_ and CO) [33,35,36]. For each pyrolysis atmosphere, a higher temperature supported a higher content of ash being retained in biochar (Table 1). At each pyrolysis temperature, biochars pyrolyzed in air limitation presented higher ash contents compared with biochars produced in CO_2_ and N_2_ flows. This is because air limitation had stronger oxidizability than CO_2_ and N_2_ flow toward biomass, thus retaining higher ash content and lower organic C content in biochars [37]. 

A high H/C ratio represents a low aromaticity and a high aliphaticity [38]. As shown in Table 1, an increasing temperature supported a decreased aliphaticity of biochar, except for the wheat straw biochar produced in air limitation (at 750 °C, it showed a sharp increase in aliphaticity). At 300~450 °C, pine sawdust biochar pyrolyzed in CO_2_ and N_2_ flows had a greater aliphaticity compared with that pyrolyzed in air limitation, while at 600~750 °C, the aliphaticity difference among the biochars from different pyrolysis atmospheres was narrowed. Meanwhile, wheat straw biochars from the three pyrolysis atmospheres showed a minimal difference in aliphaticity at 300~450 °C; however, at 600~750 °C, wheat straw biochar pyrolyzed in air limitation supported higher aliphaticity than that pyrolyzed in N_2_ and CO_2_ flows. This is because in comparison with CO_2_ and N_2_ flows, air limitation preferred to oxidize the aliphatic matter of lignin at 300~450 °C for pine sawdust; meanwhile, at 600~750 °C, the aliphatic matter of lignin could be decomposed in all studied pyrolysis atmospheres for pine sawdust [1,39,40]. Differently, the hemicellulose containing aliphatic matter with low thermostability was able to decompose in all three studied atmospheres at 300~450 °C for wheat straw [33]. A lower (N+O)/C value represents a lower polarity [17]. Table 1 presented that with the temperature being increased from 300 to 600 °C, the (N+O)/C value of biochar decreased, then it was increased at 750 °C. Pine sawdust biochars pyrolyzed in air limitation presented a higher polarity than those pyrolyzed in N_2_ and CO_2_ flows. Differently, wheat straw biochars presented the polarity orders of CO_2_ flow ≈ N_2_ flow < air limitation at 600~750 °C and N_2_ flow < air limitation < CO_2_ flow at 300~450 °C. The mechanisms of pyrolysis conditions controlling the polarity of biochar were identical to the mechanisms of pyrolysis conditions controlling the O content of biochar mentioned above.

### 3.2. BDOC Content of Biochar

**Figure 1:**
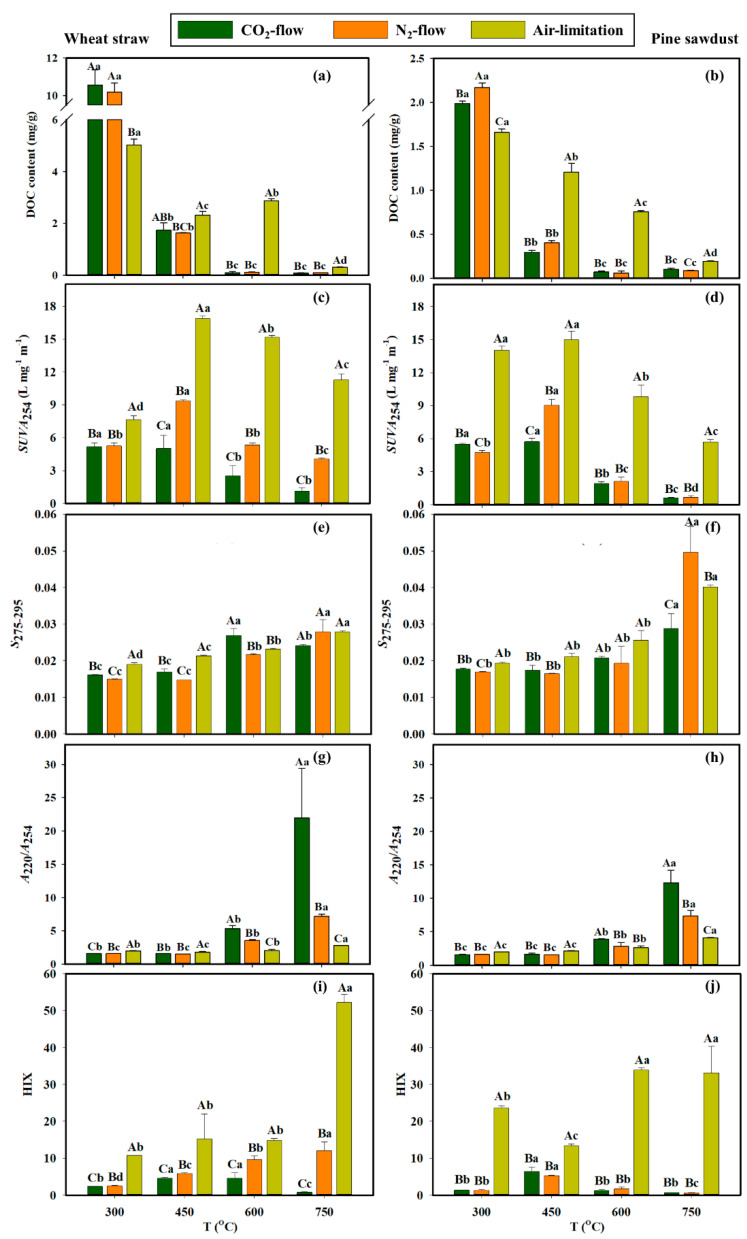
DOC content (**a**,**b**), UV-vis (**c**–**h**), and fluorescence spectral (**i**,**j**) indexes of BDOC samples from pyrolysis under different atmospheres and temperatures. Different uppercase letters indicate the significant difference in the spectral index among different pyrolysis atmospheres, and different lowercase letters indicate the significant differences in the spectral index among different pyrolysis temperatures (Tukey test, *p* < 0.05).

The influences of pyrolysis atmospheres and temperatures on the BDOC content of biochar are shown in Figure 1a,b. With the temperature being increased, the BDOC content of biochar generally reduced regardless of feedstock types and pyrolysis atmosphere. The BDOC content of biochar from 300 °C pyrolysis was about 110 times that from 750 °C pyrolysis. This result was consistent with the previous studies which suggested that the improved pyrolysis temperature could decrease the BDOC content of biochar (e.g., dewatered sludge biochar, fruit peel biochar, manure biochar, *Typha orientalis* biochar) [11,41]. BDOC consists of many volatile substances that re-agglomerate and remain in the pore structures of biochar during the procedure of pyrolysis [21]. The increasing pyrolysis temperature enhances the gasification of the volatile substances in biochar, thus decreasing the BDOC content [11]. Furthermore, Figure 1a,b show that the BDOC content of biochar could vary with the feedstock types. The BDOC content of wheat straw biochar (0.08–10.54 mg/g) was higher than that of pine sawdust biochar (0.06–2.17 mg/g) under a same pyrolysis condition, possibly due to the lignin content of pine sawdust being higher than that of wheat straw (Table S1 from Supplementary Materials). Liu et al. [42] demonstrated that the BDOC content of biochar from furfural residues was lower than that from other feedstock types (peanut shell, cotton straw, and traditional Chinese medicine residues). This was because the lignin content of furfural residues was higher than that of other feedstock types, and lignin was apt to produce rich graphitized structures during pyrolysis, which was not conducive to the formation of BDOC [14,42]. 

At each pyrolysis temperature, an insignificant difference was found in BDOC content between the biochars pyrolyzed in N_2_ and CO_2_ flows (Figure 1a,b). However, at 300 °C, the BDOC content in biochars pyrolyzed in N_2_ and CO_2_ flows was significantly higher compared with biochar pyrolyzed in air limitation (*p* < 0.05); an opposite trend was presented at 450–750 °C. A previous study has reported that the higher H/C value and (O+N)/C value of biochar indicate a lower degree of carbonization which supported biochar to release more BDOC [21,43]. This study found that, at 300 °C, the H/C and (O+N)/C values of biochar pyrolyzed in air limitation were lower compared with biochars pyrolyzed in CO_2_ and N_2_ flows (Table 1), indicating that biochar pyrolyzed in air limitation had a higher carbonization degree compared with biochar pyrolyzed in N_2_ and CO_2_ flows. Therefore, biochar pyrolyzed in air limitation contained less BDOC. However, at 450–750 °C, the H/C and (O+N)/C values of biochar pyrolyzed in N_2_ and CO_2_ flows were lower compared with biochar pyrolyzed in air limitation (Table 1), thereby biochar pyrolyzed in N_2_ and CO_2_ flows contained less BDOC. 

### 3.3. Spectral Characteristics of BDOC 

UV-vis indexes of BDOC samples are shown in Figure 1c–h. SUVA_254_ can represent the aromaticity of DOC (higher SUVA_254_ value indicates higher aromaticity of DOC) [25]. For all BDOC samples, their SUVA_254_ values initially increased with the pyrolysis temperature increasing from 300 °C to 450 °C, and then decreased at 750 °C (Figure 1c,d). Previous publications reported that organic components (e.g., cellulose and hemicellulose) in biomass can be degraded into aromatic substances and remain in biochar at a specific temperature (e.g., 500 °C), which increased the aromaticity of BDOC; however, when the pyrolysis temperature exceeded a specific temperature, the aromatic substances were decomposed into smaller compounds, which led to a reduction in BDOC aromaticity [13,44]. The SUVA_254_ value of wheat straw BDOC (1.14–16.86) was higher than that of pine sawdust BDOC (0.62–14.97) under a same pyrolysis condition (Figure 1c,d), because wheat straw contains more cellulose and hemicellulose (Table S1 from Supplementary Materials) with low thermostability which is easily decomposed into aromatic compounds during pyrolysis, while pine sawdust contains more lignin with high thermostability which is difficult to decompose during pyrolysis [45]. In Figure 1c,d, BDOC in biochar pyrolyzed in air limitation presented a significantly higher SUVA_254_ value compared with BDOC in biochar pyrolyzed in N_2_ and CO_2_ flows, and BDOC in biochar pyrolyzed in N_2_ flow generally presented a higher SUVA_254_ value compared with biochar pyrolyzed in CO_2_ flow. This is possibly due to air limitation preferring to oxidize the biomass into phenols, and CO_2_ preferring to decompose the biomass into water-insoluble aliphatics [33,34].

Normally, a smaller molecular weight of DOC shows a larger S_275–295_ [26]. With an increase in pyrolysis temperature, the S_275–295_ value of BDOC showed an upward trend (Figure 1e,f), indicating that higher temperatures preferred to form BDOC with lower molecular weight. This finding was consistent with the results previously reported [11]. This was because the aromatic components of large molecules in biochar gradually decompose into small molecules with an increasing temperature, resulting in a gradual decrease in the molecular weight of BDOC [16,29]. At 750 °C, the S_275–295_ values of pine sawdust BDOC (0.029–0.050) were significantly greater than those of wheat straw BDOC (0.024–0.028) (Figure 1e,f), indicating that pine sawdust BDOC had a smaller molecular weight than wheat straw BDOC at 750 °C. This was because lignin content in pine sawdust was higher than that in wheat straw (Table S1 from Supplementary Materials), and lignin was decomposed into low-molecular-weight organic compounds and retained in biochar at a high pyrolysis temperature [14,46]. In addition, at 300–450 °C, the S_275–295_ of BDOC samples from the three pyrolysis atmospheres indicated that the molecular weight of BDOC presented the sequence of air limitation < CO_2_ flow ≈ N_2_ flow at a low pyrolysis temperature (300 °C or 450 °C) (Figure 1e,f). However, at 600–750 °C, the S_275–295_ values of BDOC from different pyrolysis atmospheres showed a nonuniform change rule. This was because air limitation containing O_2_ can oxidize the biomass into low-molecular-weight compounds at a relatively low pyrolysis temperature (e.g., 300 °C and 450 °C), whereas biomass in CO_2_ and N_2_ can only be decomposed intensively at a relatively high temperature (e.g., 600 °C and 750 °C) [33,34,35].

A larger value of A_220_/A_254_ indicated a smaller polarity of DOC, and vice versa [24]. For each pyrolysis atmosphere, the A_220_/A_254_ values of BDOC were supported by a higher pyrolysis temperature (Figure 1g,h), which indicated that a high pyrolysis temperature supported a low polarity of BDOC, owing to the fact that the higher pyrolysis temperature facilitated the decomposition of polar organic components of biomass into gases and volatile matters (such as CO_2_ and CH_4_) [47,48]. At 600–750 °C, the A_220_/A_254_ value of BDOC from CO_2_-flow pyrolysis (wheat straw: 5.34 and 21.98; pine sawdust: 3.91 and 12.33) was larger than that from N_2_-flow pyrolysis (wheat straw: 3.53 and 7.15; pine sawdust: 2.82 and 7.39) and air-limitation pyrolysis (wheat straw: 2.04 and 2.79; pine sawdust: 2.65–4.09), indicating that the polarity of BDOC from different pyrolysis atmospheres presented the sequence of air limitation > N_2_ flow > CO_2_ flow. The phenomenon was related to the polarity of biochar. A higher (O+N)/C ratio represents a stronger polarity of biochar [18]. Table 1 showed that the polarity presented the following sequence: air limitation > N_2_ flow > CO_2_ flow at 600–750 °C, owing to the fact that hemicellulose in biomass was oxidized in air limitation more intensively than that in N_2_ and CO_2_ flows at 600–750 °C, facilitating the generation of polar substances from biomass and remaining in biochar [32,49].

A fluorescence index, HIX, of BDOC was shown in Figure 1i,j. HIX reflects the humification of BDOC and a greater HIX value indicated a higher humification [29]. The humification (HIX) of BDOC changed irregularly with an increase in pyrolysis temperature (Figure 1i,j), which was possibly due to the humification (HIX) of BDOC being strongly dependent on the pyrolysis atmospheres rather than a pyrolysis temperature. In Figure 1i, the HIX value of wheat straw BDOC from different pyrolysis atmospheres presented the following sequence: air limitation (10.77–52.21) > N_2_ flow (2.51–12.09) > CO_2_ flow (0.74–4.61). The HIX value of pine sawdust BDOC produced in different pyrolysis atmospheres presented the following sequence: air limitation (13.38–33.88) > CO_2_ flow (0.65–6.45) ≈ N_2_ flow (0.56–5.26) (Figure 1j), suggesting that BDOC in biochar pyrolyzed in air limitation had a higher humification compared with biochar pyrolyzed in N_2_ and CO_2_ flows.

### 3.4. Organic Components Analysis of BDOC Using EEM-PARAFAC Modeling

**Figure 2:**
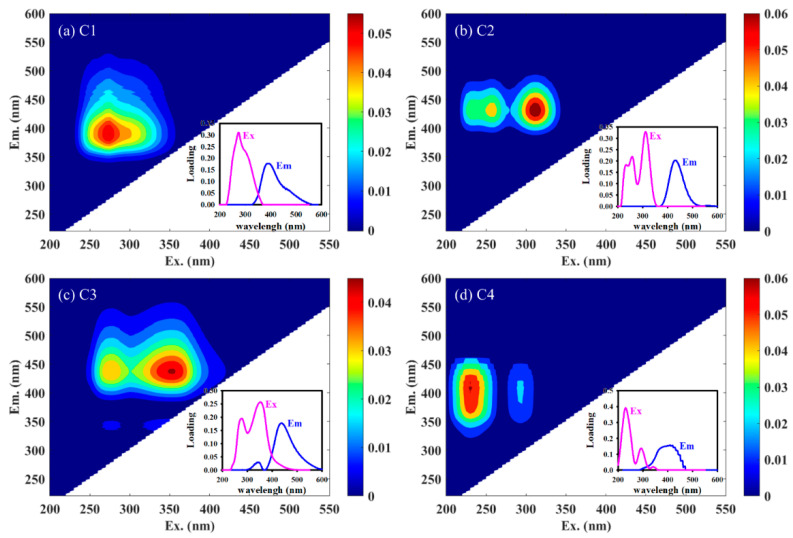
Four fluorescence components ((**a**) C1, (**b**) C2, (**c**) C3, and (**d**) C4) and their spectral loadings of BDOC were obtained using EEM-PARAFAC analysis.

Four organic components were identified after EEM-PARAFAC modeling in this study, as shown in Figure 2. C1 had a maximum Ex wavelength of 275 nm and a maximum Em wavelength of 390 nm, which represents a typical humic-like substance [50]. C2 had two Ex/Em peaks located at 255/432 nm and 310/432 nm, respectively, which indicated humic-like substances with low aromaticity and molecular weight [16]. C3 had the maximum primary Ex wavelength at 275 nm, the maximum secondary Ex wavelength at 355 nm, and the maximum Em wavelength at 438 nm, indicating that C3 represented a humic-like substance (hydrophobic component with large molecular weight) consisting of phenols and other pyrolysis products with aromatic structure [51,52]. There was an Ex/Em peak for C4 at 230/410 nm, which represents a typical fulvic-like substance [52]. The EEM-PARAFAC modeling suggested that the organic component of BDOC was dominated by humic- and fulvic-like substances [22].

**Figure 3:**
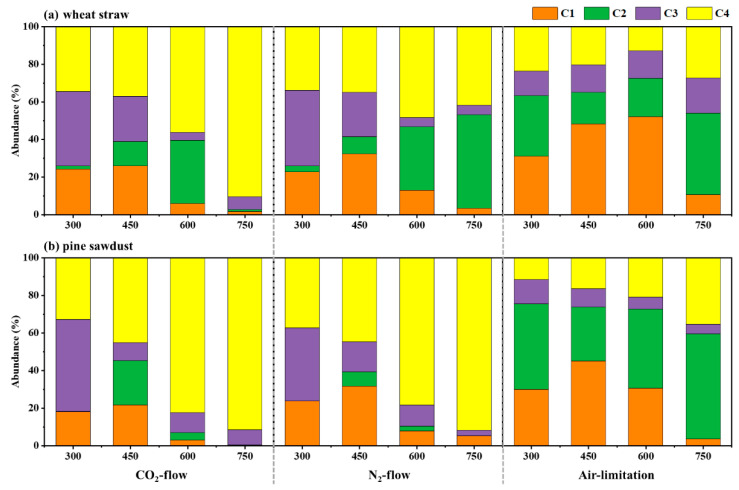
Relative abundance of fluorescent components in (**a**) wheat straw and (**b**) pine sawdust BDOC derived from different pyrolysis atmospheres at different pyrolysis temperatures.

Figure 3 presents the relative abundances of the four organic components in BDOC. For wheat straw (Figure 3a), the humic-like substances abundance (C1 + C2 + C3) and the fulvic-like substances abundance (C4) of BDOC from CO_2_-flow pyrolysis decreased from 0.66 to 0.10 and increased from 0.34 to 0.90, respectively, with the increasing pyrolysis temperature. Differently, the abundances of humic-like substances (C1 + C2 + C3) (N_2_-flow: 0.51–0.66; CO_2_-flow: 0.73–0.87) and fulvic-like substances (C4) (N_2_-flow: 0.34–0.42; CO_2_-flow: 0.12–0.27) of BDOC from N_2_-flow and air-limitation pyrolysis had a little change with the increasing pyrolysis temperature. For pine sawdust (Figure 3b), the abundances of humic-like substances and the fulvic-like substances of BDOC from each pyrolysis atmosphere exhibited a decreasing and increasing trend, respectively, with the increasing pyrolysis temperature. The abundance of humic-like substance of BDOC from different pyrolysis atmospheres approximately followed the order of air limitation (0.65–0.89) > N_2_ flow (0.08–0.66) ≈ CO_2_ flow (0.08–0.67), and the abundance of fulvic-like substance in BDOC approximately followed the order of CO_2_ flow (0.33–0.91) ≈ N_2_ flow (0.34–0.92) > air limitation (0.11–0.35). As shown in Figure 3, humic-like substances primarily existed in BDOC from low pyrolysis temperatures and air-limitation atmospheres, while fulvic-like substances primarily existed in BDOC from CO_2_-flow and N_2_-flow pyrolysis atmospheres at high pyrolysis temperatures. Hence, our result suggested that the BDOC from air-limitation pyrolysis and the BDOC from O_2_-free pyrolysis (CO_2_ and N_2_ atmospheres) have a significant difference in organic components.

### 3.5. Quantitative Relationships between Characteristics of BDOC and Properties of Biochar

**Figure 4:**
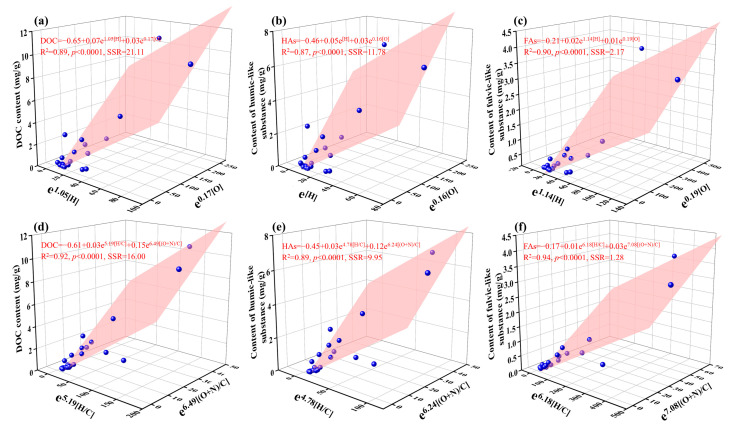
The quantitative relationship between the characteristics of BDOC (bulk DOC (**a**,**d**), humic-like substances (HAs) (**b**,**e**), and fulvic-like substance (FAs) (**c**,**f**) contents) and the exponential properties (H and O contents (**a**–**c**), H/C and (O+N)/C) (**d**–**f**) of biochar. SSR indicates sum of squared residual.

In this study, the contents of humic-like substances (C1, C2, and C3) and fulvic-like substances (C4) in BDOC were calculated according to the relative abundance of fluorescent components and their DOC content (organic component content = relative abundance of each component × DOC content). The quantitative relationships between the characteristics of BDOC (bulk DOC, humic-like, and fulvic-like substances contents) and the properties of biochar (H and O contents, H/C and (O+N)/C, volatile matter content) were established using the linear equation and the exponential equation (Table S3 from Supplementary Materials). The correlation coefficient (*R*^2^), significance index (*p*), and the sum of squared residual (SSR) (Table S3 from Supplementary Materials) all indicated that the exponential equation could better describe the relationships between the characteristics (bulk DOC and organic components contents) of BDOC and the properties of biochar (H and O content, H/C and (O+N)/C, and volatile matter) than the linear equation. For instance, the *R*^2^ values of the exponential equation (H content: 0.62; H/C atomic ratio: 0.79) between DOC content and the properties (e.g., H content, and H/C atomic ratio) of biochar were larger than those of the linear equation (H content: 0.47; H/C atomic ratio: 0.63). Additionally, the *R*^2^ values of the exponential equation (H content: 0.61 and 0.62; H/C atomic ratio: 0.76 and 0.81) between the organic component contents (the contents of humic-like and fulvic-like substances) and the properties (e.g., H content and H/C ratio) of biochar were greater than those of the linear equation (H content: 0.47 and 0.44; H/C ratio: 0.63 and 0.58). Meanwhile, the *p* and SSR values of the exponential equation (*p* < 0.0001, SSR = 23.80) between the DOC content and volatile matter content of biochar were lower than those of the linear equation (*p* = 0.001, SSR = 69.97). Accordingly, the exponential forms of the parameters (H and O contents, H/C and (O+N)/C, and volatile matter content) of biochar were used to perform the multiple linear regression to quantitatively evaluate the characteristics of BDOC (the contents of bulk DOC, humic-like substances, and fulvic-like substances). The quantitative results are shown in Figure 4, and the *R*^2^ values of the multiple linear equations between the characteristics of BDOC (the contents of bulk DOC, humic-like and fulvic-like substances) and the exponential forms of H and O contents of biochar were 0.87~0.90. The *R*^2^ values of the fitting multiple linear equations between the characteristics of BDOC (the contents of bulk DOC and humic-like and fulvic-like substances) and the exponential forms of H/C and (O+N)/C atomic ratio of biochar were 0.89~0.94. Additionally, the *p*-values of these quantitative relationships were less than 0.0001. The results showed that the exponential element content (H and O content) and element atomic ratio (H/C and (O+N)/C) were applicable to quantitatively predict DOC content and the proportion of organic components, and the specific equations are shown in Figure 4.

### 3.6. The Classification of BDOC Using EEM-SOM and PARAFAC-SOM Analysis

**Figure 5:**
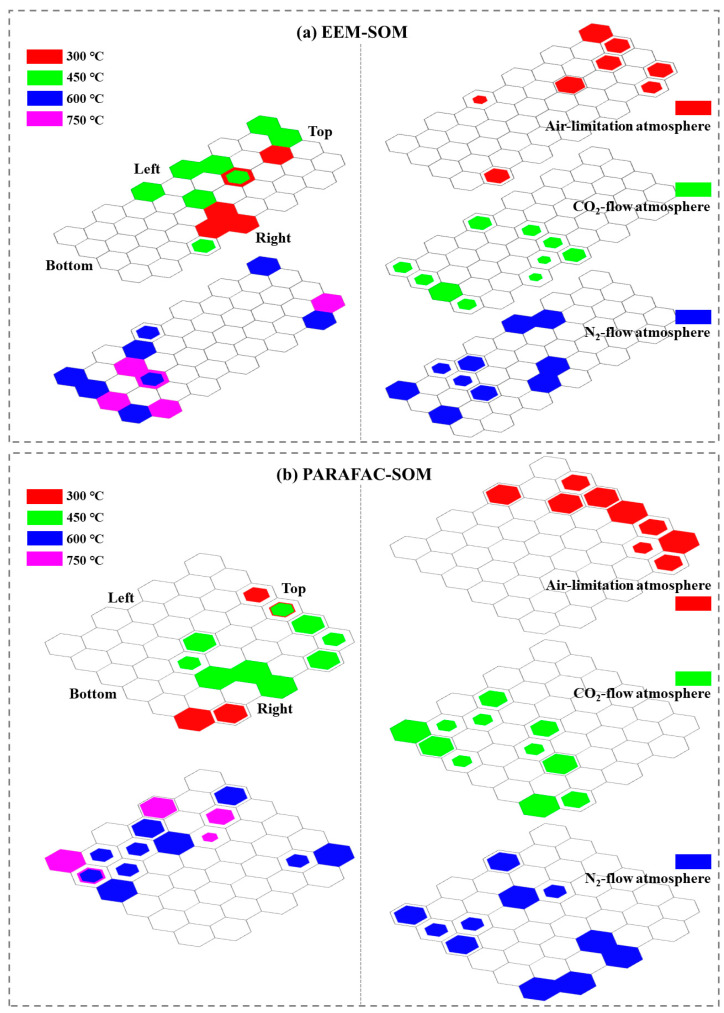
(**a**) Multiple hits map of EEM-SOM analysis, (**b**) multiple hits map of PARAFAC-SOM analysis.

The unified distance matrix (U-matrix) and the mapping distribution map (Figure S2 from Supplementary Materials) presented the EEM distribution characteristics of BDOC samples in each neuron that can be obtained by SOM training. The U-matrix describes the degree of similarity (measured by Euclidean distance) between neurons as additional regular hexagonal elements [23]. In the U-matrix, the redder color (the larger the distance value) indicated a greater difference in fluorescence properties between neighboring neurons, which was helpful to determine the borders of the clusters [26]. In the vertical direction, the sample map (Figure S2 from Supplementary Materials) comprised two parts. In the multiple hits map (Figure 5a (left)), BDOC samples pyrolyzed at 300 °C (red) and 450 °C (green), located on the top of the map, could be classified into a cluster, while BDOC samples pyrolyzed by 600 °C (blue) and 750 °C (purple), mainly located on the bottom of the map, could be classified into another cluster. In Figure 5a (right), BDOC samples from air-limitation pyrolysis (red) were distributed on the top of the map could be classified into a cluster, while BDOC samples from CO_2_-flow (green) and N_2_-flow (blue) pyrolysis were distributed on the bottom of the map and could be classified into another cluster. In addition, the fluorescence intensity of the samples distributed on the top was higher than that on the bottom (Figure S3 from Supplementary Materials), which indicated that substances with higher fluorescence intensity occurred in low-temperature pyrolysis and air-limitation pyrolysis BDOC. These results were in line with those of the PARAFAC analysis (Section 3.4).

In order to effectively distinguish the BDOC according to the organic compositions, a new PARAFAC-SOM model was constructed by using the composition abundance data of organic components. The PARAFAC-SOM model can generalize and visualize the variation law of fluorescence component abundance among BDOC samples with different pyrolysis temperatures and atmospheres. The SOM visual output map based on the PARAFAC-SOM model was shown in Figure S4 (from Supplementary Materials). In the multiple hits map (Figure 5b (left)), BDOC from low pyrolysis temperatures and high pyrolysis temperatures was mainly distributed on the right and the left, respectively, of the map. In the multiple hits map (Figure 5b (right)), BDOC from air-limitation pyrolysis and O_2_-free (CO_2_- and N_2_-flow) pyrolysis was mainly distributed on the top and the bottom, respectively, of the map. Meanwhile, the humic-like substances of each neuron in the map decreased from upper to bottom, while fulvic-like substances increased from upper to bottom (Figure S5 from Supplementary Materials). According to the analysis in Figure 5b, C1 and C2 components played a dominant role in BDOC of air-limitation pyrolysis, and C3 components played a dominant role in BDOC of N_2_- and CO_2_-flow pyrolysis at 300–450 °C, and C4 components played a dominant role in BDOC of N_2_- and CO_2_-flow pyrolysis at 600–750 °C. In conclusion, PARAFAC-SOM modeling showed that humic-like substances were the main organic components in BDOC from air-limitation pyrolysis, and fulvic-like substances were the dominant organic components in BDOC from CO_2_- and N_2_-flow pyrolysis at 600–750 °C, and humic-like substances were dominant in BDOC samples from CO_2_- and N_2_-flow atmosphere at 300–450 °C. This was consistent with the results of the EEM-PARAFAC analysis (Figure 5b). These results indicated that the organic compositions of BDOC produced in O_2_-free pyrolysis (CO_2_- and N_2_-flow) and BDOC produced in air-limitation were significantly different, thereby they likely have different environmental fates and effects.

## 4. Conclusions

This work deeply explored the various characteristics of BDOC from three pyrolysis atmosphere types (including N_2_ flow, CO_2_ flow, and air limitation) at 300–750 °C. The UV-vis and fluorescence spectrums showed that BDOCs from the three pyrolysis atmospheres present significant difference in releasable content (BDOC content at 450–750 °C, CO_2_ flow: 0.07–1.74 mg/g; N_2_ flow: 0.06–1.63 mg/g; air limitation: 0.19–2.88 mg/g), aromaticity (CO_2_ flow: 0.62–5.71; N_2_ flow: 0.67–9.34; air limitation: 5.70–16.86), polarity (A_220_/A_254_ at 600–750 °C, CO_2_ flow: 3.91–21.98; N_2_ flow: 2.82–7.39; air limitation:2.04–4.09), molecular size (S_275–295_ at 300–450 °C, CO_2_: 0.0161–0.0178; N_2_: 0.0147–0.0169; air limitation: 0.0190–0.0213), and organic compositions (including humic-like substances and fulvic-like substances). The exponential parameters (H and O contents, (O+N)/C and H/C ratios) of biochar could be effectively used to speculate the content of BDOC and its organic component contents in biochar. Additionally, the self-organizing maps could effectively visualize the categories of fluorescence intensity and components of BDOC from different pyrolysis atmospheres and temperatures. This study demonstrated that the BDOC derived from different atmosphere pyrolysis of biomass possibly had different environmental fates and effects, and the release amount and organic composition of BDOC can be quantitatively predicted based on the elemental compositions of biochar.

## Figures and Tables

**Table 1:** Selected elemental compositions and properties of biochar.

	Atmosphere	Temperature (℃)	C (wt.%)	N (wt.%)	H (wt.%)	O (wt.%)	H/C	(O+N)/C	Volatile Matter (wt.%)	Fixed C (wt.%)	Ash (wt.%)
Wheat straw biochars	CO_2_ flow	300	43.31 ± 0.23	1.23 ± 0.03	3.40 ± 0.02	32.45 ± 0.32	0.94 ± 0.00	0.59 ± 0.01	67.69 ± 3.28	12.70 ± 3.33	19.61 ± 0.08
	CO_2_ flow	450	47.26 ± 0.96	1.06 ± 0.02	2.60 ± 0.25	27.00 ± 0.73	0.62 ± 0.00	0.38 ± 0.11	55.56 ± 3.51	22.37 ± 3.53	22.08 ± 0.25
	CO_2_ flow	600	58.78 ± 0.42	0.90 ± 0.02	1.73 ± 0.01	14.44 ± 0.59	0.35 ± 0.00	0.20 ± 0.01	55.99 ± 2.23	19.87 ± 2.01	24.14 ± 0.22
	CO_2_ flow	750	44.36 ± 0.18	0.91 ± 0.03	1.33 ± 0.01	15.41 ± 0.04	0.36 ± 0.00	0.28 ± 0.00	47.04 ± 4.24	14.96 ± 4.21	37.99 ± 0.21
	N_2_ flow	300	52.03 ± 0.26	1.12 ± 0.03	4.43 ± 0.02	27.27 ± 0.15	1.02 ± 0.00	0.41 ± 0.00	55.56 ± 3.22	29.30 ± 3.22	15.14 ± 0.14
	N_2_ flow	450	55.97 ± 0.11	0.97 ± 0.04	3.21 ± 0.01	19.17 ± 0.12	0.69 ± 0.00	0.27 ± 0.00	47.71 ± 5.00	31.61 ± 5.01	20.68 ± 0.05
	N_2_ flow	600	57.10 ± 0.41	0.84 ± 0.03	2.18 ± 0.02	16.60 ± 0.51	0.46 ± 0.00	0.23 ± 0.01	35.08 ± 7.45	41.64 ± 7.52	23.28 ± 0.16
	N_2_ flow	750	55.73 ± 0.38	0.65 ± 0.18	1.55 ± 0.18	17.75 ± 0.71	0.35 ± 0.00	0.32 ± 0.14	28.49 ± 5.15	47.19 ± 5.16	24.32 ± 0.21
	Air limitation	300	49.16 ± 0.09	1.18 ± 0.01	3.72 ± 0.40	28.12 ± 0.71	0.85 ± 0.00	0.38 ± 0.13	59.68 ± 5.11	22.47 ± 4.55	17.85 ± 0.55
	Air limitation	450	46.30 ± 0.26	1.07 ± 0.02	2.61 ± 0.01	22.41 ± 0.58	0.68 ± 0.00	0.38 ± 0.01	51.93 ± 3.91	20.46 ± 3.87	27.61 ± 0.29
	Air limitation	600	37.21 ± 0.04	0.78 ± 0.04	1.86 ± 0.00	16.60 ± 0.47	0.60 ± 0.00	0.35 ± 0.01	42.22 ± 3.71	14.23 ± 3.42	43.56 ± 0.46
	Air limitation	750	7.39 ± 0.02	0.27 ± 0.02	0.84 ± 0.00	8.22 ± 0.29	1.36 ± 0.00	0.87 ± 0.03	11.79 ± 0.05	4.94 ± 0.30	83.27 ± 0.25
Pine sawdust biochars	CO_2_ flow	300	61.90 ± 0.29	0.18 ± 0.03	4.75 ± 0.02	26.36 ± 0.32	0.92 ± 0.00	0.32 ± 0.01	61.03 ± 4.27	32.17 ± 4.29	6.81 ± 0.04
	CO_2_ flow	450	72.56 ± 0.39	0.23 ± 0.03	3.18 ± 0.02	14.00 ± 0.46	0.53 ± 0.00	0.15 ± 0.01	33.02 ± 0.96	56.96 ± 0.88	10.03 ± 0.06
	CO_2_ flow	600	76.31 ± 0.02	0.23 ± 0.03	1.97 ± 0.00	9.98 ± 0.11	0.31 ± 0.00	0.10 ± 0.00	26.60 ± 3.49	61.92 ± 3.61	11.50 ± 0.09
	CO_2_ flow	750	68.20 ± 0.07	0.26 ± 0.02	1.40 ± 0.00	10.60 ± 0.16	0.25 ± 0.00	0.15 ± 0.00	23.28 ± 2.14	60.18 ± 2.26	16.53 ± 0.12
	N_2_ flow	300	62.01 ± 0.18	0.19 ± 0.04	4.36 ± 0.01	26.95 ± 0.19	0.84 ± 0.00	0.33 ± 0.00	60.57 ± 2.48	32.94 ± 2.46	6.49 ± 0.05
	N_2_ flow	450	71.34 ± 0.24	0.21 ± 0.02	3.28 ± 0.01	15.51 ± 0.25	0.55 ± 0.00	0.17 ± 0.00	37.86 ± 1.09	52.49 ± 1.06	9.66 ± 0.05
	N_2_ flow	600	75.79 ± 0.04	0.19 ± 0.01	2.17 ± 0.00	10.42 ± 0.07	0.34 ± 0.00	0.11 ± 0.00	27.25 ± 1.16	61.32 ± 1.12	11.44 ± 0.04
	N_2_ flow	750	75.67 ± 0.08	0.22 ± 0.03	1.43 ± 0.00	10.63 ± 0.10	0.23 ± 0.00	0.11 ± 0.00	22.96 ± 1.17	64.98 ± 1.22	12.06 ± 0.14
	Air limitation	300	59.31 ± 0.19	0.30 ± 0.01	2.72 ± 0.01	28.15 ± 0.21	0.55 ± 0.00	0.36 ± 0.00	53.60 ± 3.35	36.89 ± 3.37	9.51 ± 0.02
	Air limitation	450	58.37 ± 0.16	0.37 ± 0.02	2.34 ± 0.01	20.39 ± 0.21	0.48 ± 0.00	0.27 ± 0.00	43.42 ± 4.08	38.05 ± 4.04	18.53 ± 0.04
	Air limitation	600	58.40 ± 0.35	0.23 ± 0.02	1.63 ± 0.01	13.53 ± 0.48	0.34 ± 0.00	0.18 ± 0.01	28.82 ± 2.57	44.97 ± 2.31	26.21 ± 0.27
	Air limitation	750	34.27 ± 0.16	0.14 ± 0.01	0.70 ± 0.00	11.71 ± 0.48	0.24 ± 0.00	0.26 ± 0.01	22.38 ± 0.31	24.43 ± 0.43	53.19 ± 0.37

## Data Availability

Not applicable.

## Supplementary Materials

The following supporting information can be downloaded at: https://www.mdpi.com/article/10.3390/molecules28052247/s1. Figure S1: UV-vis spectra of BDOC derived from different pyrolysis atmospheres at different pyrolysis temperatures; Figure S2: (a) U-matrix, (b) SOM visualization map, (c) single hit map of EEM-SOM analysis; Figure S3: Fluorescence spectrum of reference EEM in typical neurons; Figure S4: (a) U-matrix, (b) SOM visualization map, (c) single hit map of PARAFAC-SOM analysis; Figure S5: The abundance of reference components in typical neurons. Table S1: Main organic components of different biomasses; Table S2: F_max_ of different fluorescence components in BDOC; Table S3: Fitting relationship between the characteristics of BDOC (bulk DOC, humic-like substance, and fulvic-like substance contents) and the properties of biochar. Text S1: Fluorescence excitation and emission spectrum determination and analysis of BDOC. Refs. [7,25,27,53,54,55,56,57,58] are cited in the supplementary materials.
