# Supplementary material for: Pyrolysis Atmospheres and Temperatures Co-Mediated Spectral Variations of Biochar-Derived Dissolved Organic Carbon: Quantitative Prediction and Self-Organizing Maps Analysis
Source: Molecules. 2023 Feb 28;28(5):2247. doi: 10.3390/molecules28052247 (PMC10005102; doi:10.3390/molecules28052247)
Supplement: Supplementary file 1 [file molecules-28-02247-s001.zip › molecules-2252782-supplementary.pdf]

Pyrolysis Atmospheres and Temperatures Co-Mediated Spectral Variations of Biochar-Derived Dissolved Organic Carbon: Quantitative Prediction and Self-Organizing Maps Analysis

Text S1. *Fluorescence excitation and emission spectrum determination and analysis of BDOC.*

Inner-filtering effects were minimized by diluting the BDOC concentration to 10 mg/L [S1,S2]. In this study, 60 samples were used for fluorescence determination. The fluoresce excitation and emission matrix (EEM) spectra were scanned by a fluorescence spectrophotometer (F-7000 FL, Hitachi). Excitation and emission wavelength ranges were set from 200-550 nm and 220-600 nm respectively. Excitation and emission scanning intervals were set at 5 nm and 2 nm respectively. Scan speed was set at 1200 nm/min, and excitation and emission slit widths were fixed at 10 nm. Photomultiplier detector voltage was fixed at 640V. The blank scans were performed at intervals of 10 analyses using ultrapure water. Ultrapure water blanks were measured and the EEMs were subtracted from SDOC measurements to remove Raman scattering effects. PARAFAC was used to model the dataset of fluoresce EEMs. It statistically decomposes the complex mixture of DOC fluorophores into independent components about their spectral shapes or numbers, thus allowing the estimation of the true underlying EEM spectra between the samples. The approach of PARAFAC modeling to EEMs has been described in detail in previous studies [S2,S3,S4,S5]. In this study, DOMFluor Toolbox for MATLAB was used to fit the PARAFAC model over a data set comprising all extraction samples. The correct number of components was identified by several methods of split half analysis, residual analysis, and visual inspection [S5]. PARAFAC modeling was conducted with non-negativity constraint. No outliers were identified during the analysis. The maximum fluorescence intensity was

used as the representative values for each fluorescent component [S6,S7]. In this study, four fluorescence components (C1, C2, C3, and C4) were extracted and the F_{max} was shown in **Table S3**. The percentage abundance of each component (e.g., C1%, C2%, C3%, and C4%) was calculated as a chemical composition. Split-half analysis results were consistent with the model.

Table S1. Main organic components of different biomasses

Biomasses	Cellulose (wt.%)	Hemicellulose (wt.%)	Lignin (wt.%)	References
Wheat straw	35.1	27.1	5.3	[S8]
Pine sawdust	38	15	28	[S9]

Table S2. F_{max} of different fluorescence components in BDOC

	Atmosphere	Temperature (℃)	C1	C2	C3	C4
Wheat straw derived BDOC	CO_2-flow	300	686.26±46.20	52.54±6.83	1064.50±65.03	977.66±39.69
	CO_2-flow	450	838.98±169.53	423.28±121.39	611.83±144.38	1192.33±212.63
	CO_2-flow	600	13.51±12.27	71.87±15.49	9.60±2.46	123.12±21.18
	CO_2-flow	750	1.32±2.29	0.54±0.92	3.82±2.13	52.09±14.47
	N_2-flow	300	656.78±24.96	93.67±2.90	1103.72±51.82	983.31±27.13
	N_2-flow	450	1227.90±44.79	338.49±12.66	904.33±14.44	1322.72±89.81
	N_2-flow	600	72.01±4.71	188.83±9.81	30.92±2.75	269.52±6.64
	N_2-flow	750	17.24±29.85	196.55±15.60	66.26±36.63	169.68±25.91
	Air-limitation	300	1829.21±85.22	1902.01±113.01	735.07±39.38	1397.23±98.67
	Air-limitation	450	5222.66±2982.83	1373.34±443.90	1947.90±679.28	1964.36±528.75
	Air-limitation	600	5592.01±443.89	2188.19±113.69	1541.35±65.28	1397.05±431.08
	Air-limitation	750	384.40±8.53	1579.84±38.82	678.61±8.71	992.85±21.19
Pine sawdust-derived BDOC	CO_2-flow	300	561.57±13.97	6.32±10.94	1489.55±15.30	1010.84±18.56
	CO_2-flow	450	426.89±28.88	472.83±114.84	175.02±11.73	887.91±59.35
	CO_2-flow	600	4.52±7.83	4.35±5.94	13.17±0.64	99.71±19.41
	CO_2-flow	750	0.44±0.76	0.00±0.00	5.43±3.08	69.62±8.78
	N_2-flow	300	628.02±25.18	3.87±6.70	982.90±32.48	983.58±39.22
	N_2-flow	450	628.01±2.72	152.95±13.40	330.07±9.77	886.99±8.75
	N_2-flow	600	10.35±5.11	3.11±5.38	16.20±1.58	101.61±12.51
	N_2-flow	750	5.23±7.09	0.00±0.00	0.93±1.63	69.69±19.37
	Air-limitation	300	2686.94±114.52	4057.21±277.41	1101.60±51.15	1027.03±122.77
	Air-limitation	450	4982.87±254.40	3159.61±457.95	1042.41±86.41	1812.77±308.87
	Air-limitation	600	2316.93±203.43	3760.50±35.59	492.80±30.12	1489.19±225.99
	Air-limitation	750	43.60±12.37	658.65±25.72	62.61±1.62	417.32±9.71

Table S3. Fitting relationship between the characteristics of BDOC (bulk DOC, humic-like substance, and fulvic-like substance contents) and the properties of biochar.

BDOC Characteristics (y)	Biochar compositions (x)	Linear equation				Exponential equation			
		Equation	R^2	p	SSR	Equation	R^2	p	SSR
DOC content	C	y=4.06-0.04x	0.05	0.31	183.42	-	-	-	-
	N	y=-0.66+4.36x	0.36	0.002	123.53	$y=0.002e^{6.85x}$	0.70	<0.0001	57.44
	H	y=-3.11+2.15x	0.47	0.0004	101.41	$y=0.1e^{1.05x}$	0.62	<0.0001	72.67
	O	y=-3.80+0.31x	0.51	<0.0001	94.36	$y=0.04e^{0.17x}$	0.66	<0.0001	65.60
	H/C	y=-3.31+9.59x	0.63	<0.0001	70.89	$y=0.05e^{5.19x}$	0.79	<0.0001	40.53
	(O+N)/C	y=-3.31+19.05x	0.61	<0.0001	74.68	$y=0.26e^{6.49x}$	0.68	<0.0001	60.13
	Volatile matter	y=-2.58+0.1x	0.41	0.001	71.34	$y=0.001e^{0.14x}$	0.68	<0.0001	37.90
	Fixed C	y=-4.22-0.07x	0.16	0.05	160.80	-	-	-	-
	Ash	y=2.36-0.02x	0.02	0.53	188.85	-	-	-	-
Humic-like substance	C	y=2.92-0.03x	0.06	0.27	82.81	-	-	-	-
	N	y=-0.44+2.99x	0.37	0.0016	55.18	$y=0.002e^{6.56x}$	0.69	<0.0001	27.00
	H	y=-2.06+1.46x	0.47	0.0004	46.09	$y=0.08e^{x}$	0.61	<0.0001	34.49
	O	y=-2.57+0.21x	0.52	<0.0001	42.08	$y=0.04e^{0.16x}$	0.64	<0.0001	31.40
	H/C	y=-2.21+6.51x	0.63	<0.0001	31.65	$y=0.05e^{4.78x}$	0.76	<0.0001	21.09
	(O+N)/C	y=-2.26+13.13x	0.63	<0.0001	31.79	$y=0.2e^{6.24x}$	0.67	<0.0001	28.20
	Volatile matter	y=-1.76+0.07x	0.41	0.001	31.79	$y=0.002e^{0.11x}$	0.62	<0.0001	21.71
	Fixed C	y=2.98-0.05x	0.18	0.04	71.64	-	-	-	-
	Ash	y=1.59-0.01x	0.01	0.58	86.44	-	-	-	-
Fulvic-like substance	C	y=1.14-0.01x	0.03	0.43	20.91	-	-	-	-
	N	y=-0.23+1.36x	0.31	0.05	14.78	$y=0.0003e^{7.49x}$	0.68	<0.0001	6.87
	H	y=-1.05+0.7x	0.44	0.0007	11.95	$y=0.02e^{1.14x}$	0.62	<0.0001	8.14
	O	y=-1.24+0.1x	0.46	0.0003	11.62	$y=0.01e^{0.19x}$	0.66	<0.0001	7.27
	H/C	y=-1.1+3.08x	0.58	<0.0001	9.01	$y=0.01e^{6.18x}$	0.81	<0.0001	4.00
	(O+N)/C	y=-1.04+5.92x	0.52	<0.0001	10.15	$y=0.06e^{7.08x}$	0.67	<0.0001	7.06
	Volatile matter	y=-0.82+0.03x	0.36	0.002	8.12	$y=1.46\times10^{-6}e^{0.22x}$	0.80	<0.0001	2.55
	Fixed C	y=1.24-0.02x	0.12	0.1	18.92	-	-	-	-
	Ash	y=0.77-0.01x	0.03	0.44	20.94	-	-	-	-

Note: SSR indicates the sum of squared residual.

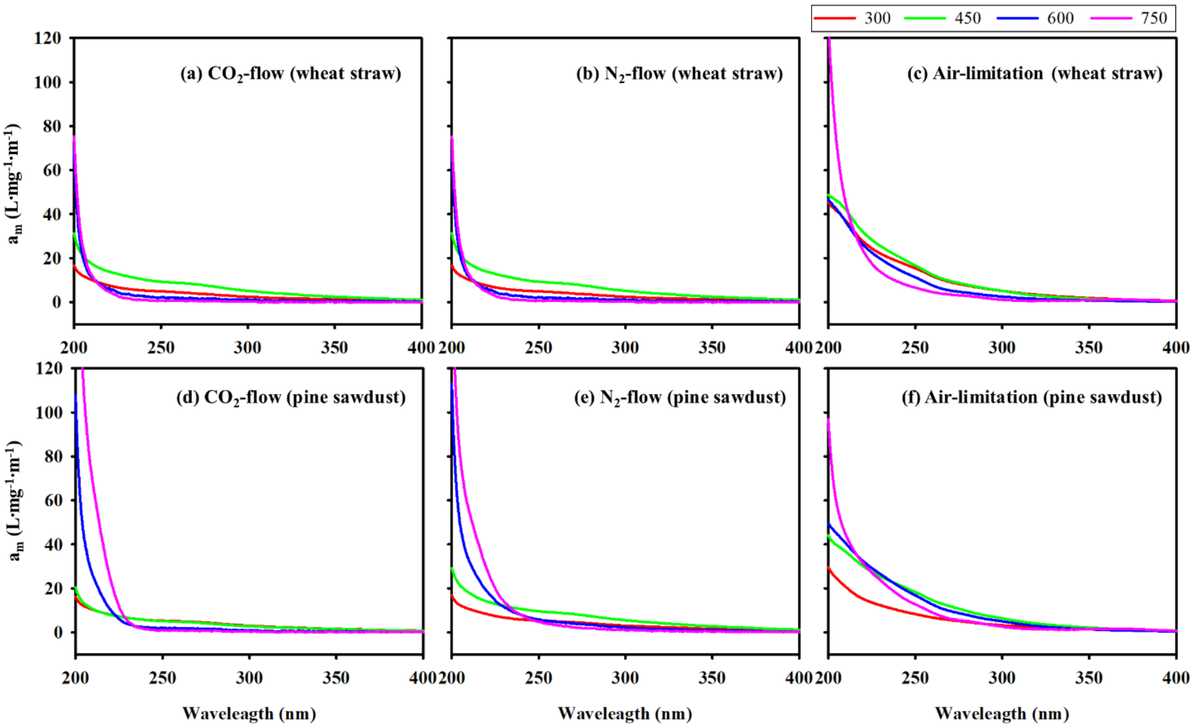

Figure S1. UV-vis spectra of BDOC derived from different pyrolysis atmospheres at different pyrolysis temperatures.

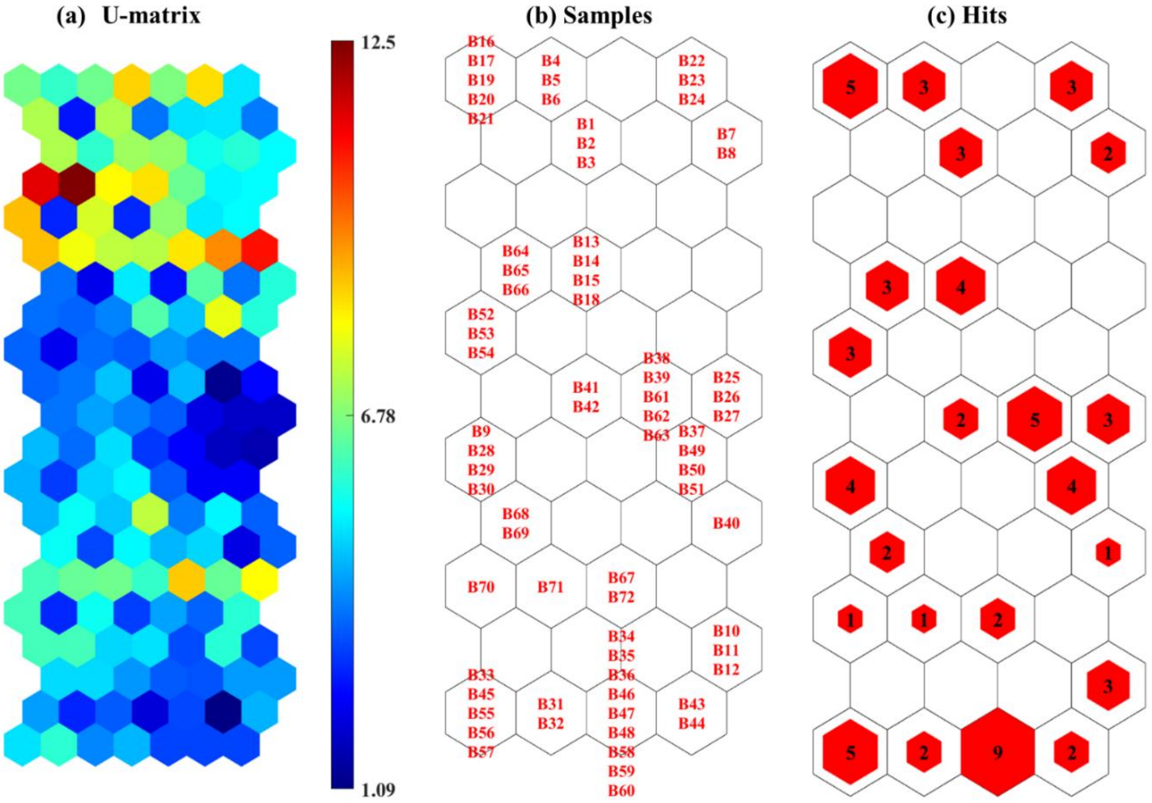

Figure S2. (a) U-matrix, (b) SOM visualization map, (c) single hit map of EEM-SOM analysis (B1-B24 indicate air-limitation atmosphere, B25-B48 indicate CO_2-flow atmosphere, B49-B72 indicate N_2-flow atmosphere; B1-B12, B25-B36 and B49-B60 indicate pine sawdust; B13-B24, B37-B48, and B61-B72 indicate wheat straw; B1-B3, B13-B15, B25-B27, B37-B39, B49-B51, and B61-B63 indicate the pyrolysis temperature of 300 ℃; B4-B6, B16-B18, B28-B30, B40-B42, B52-B54, and B64-B66 indicate 450 ℃; B7-B9, B19-B21, B31-B33, B43-B45, B55-B57, and B67-B69 indicate 600 ℃; B10-B12, B22-B24, B34-B36, B46-B48, B58-B60, and B70-B72 indicate 750 ℃)

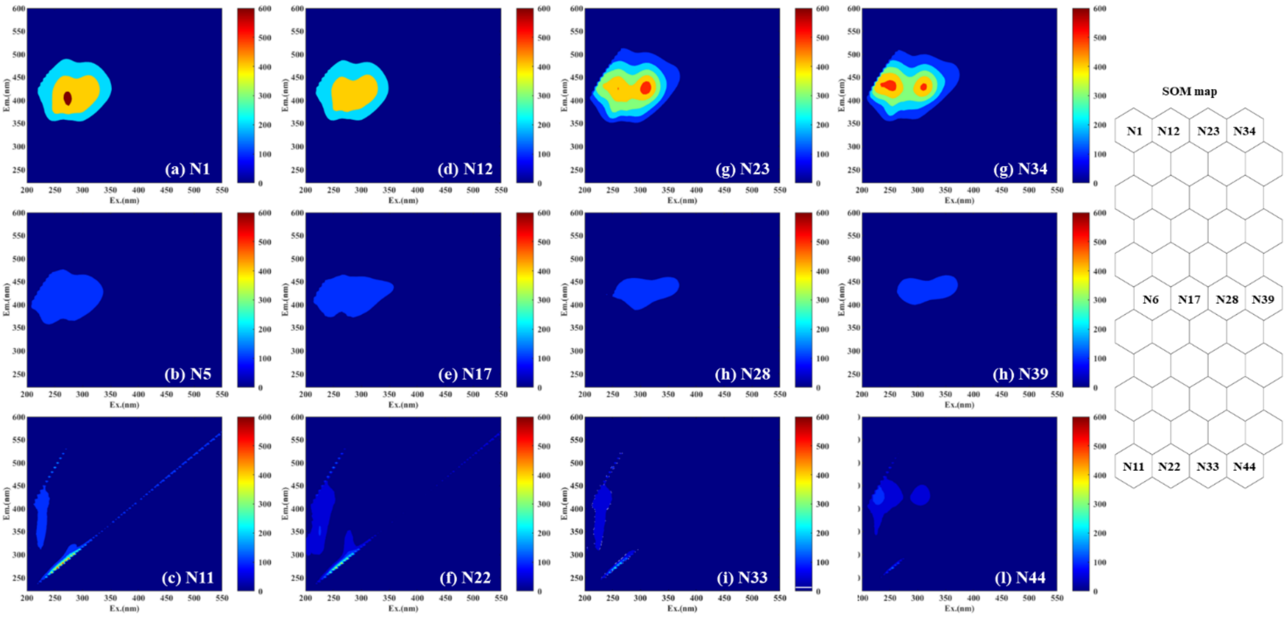

Figure S3. Fluorescence spectrum of reference EEM in typical neurons.

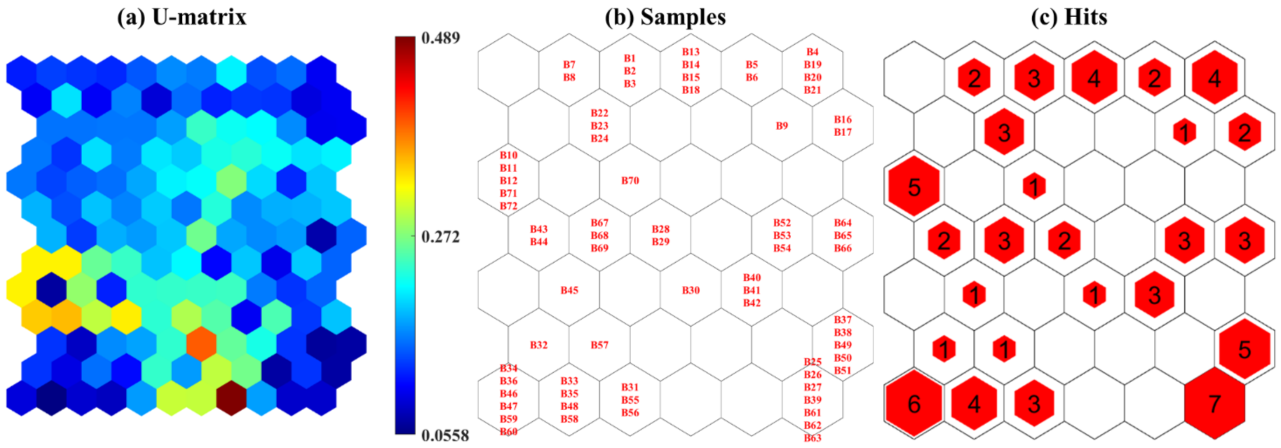

Figure S4. (a) U-matrix, (b) SOM visualization map, (c) single hit map of PARAFAC-SOM analysis. (B1-B24 indicate air-limitation atmosphere, B25-B48 indicate CO_2-flow atmosphere, B49-B72 indicate N_2-flow atmosphere; B1-B12, B25-B36 and B49-B60 indicate pine sawdust; B13-B24, B37-B48, and B61-B72 indicate wheat straw; B1-B3, B13-B15, B25-B27, B37-B39, B49-B51, and B61-B63 indicate the pyrolysis temperature of 300 ℃; B4-B6, B16-B18, B28-B30, B40-B42, B52-B54, and B64-B66 indicate 450 ℃; B7-B9, B19-B21, B31-B33, B43-B45, B55-B57, and B67-B69 indicate 600 ℃; B10-B12, B22-B24, B34-B36, B46-B48, B58-B60, and B70-B72 indicate 750 ℃)

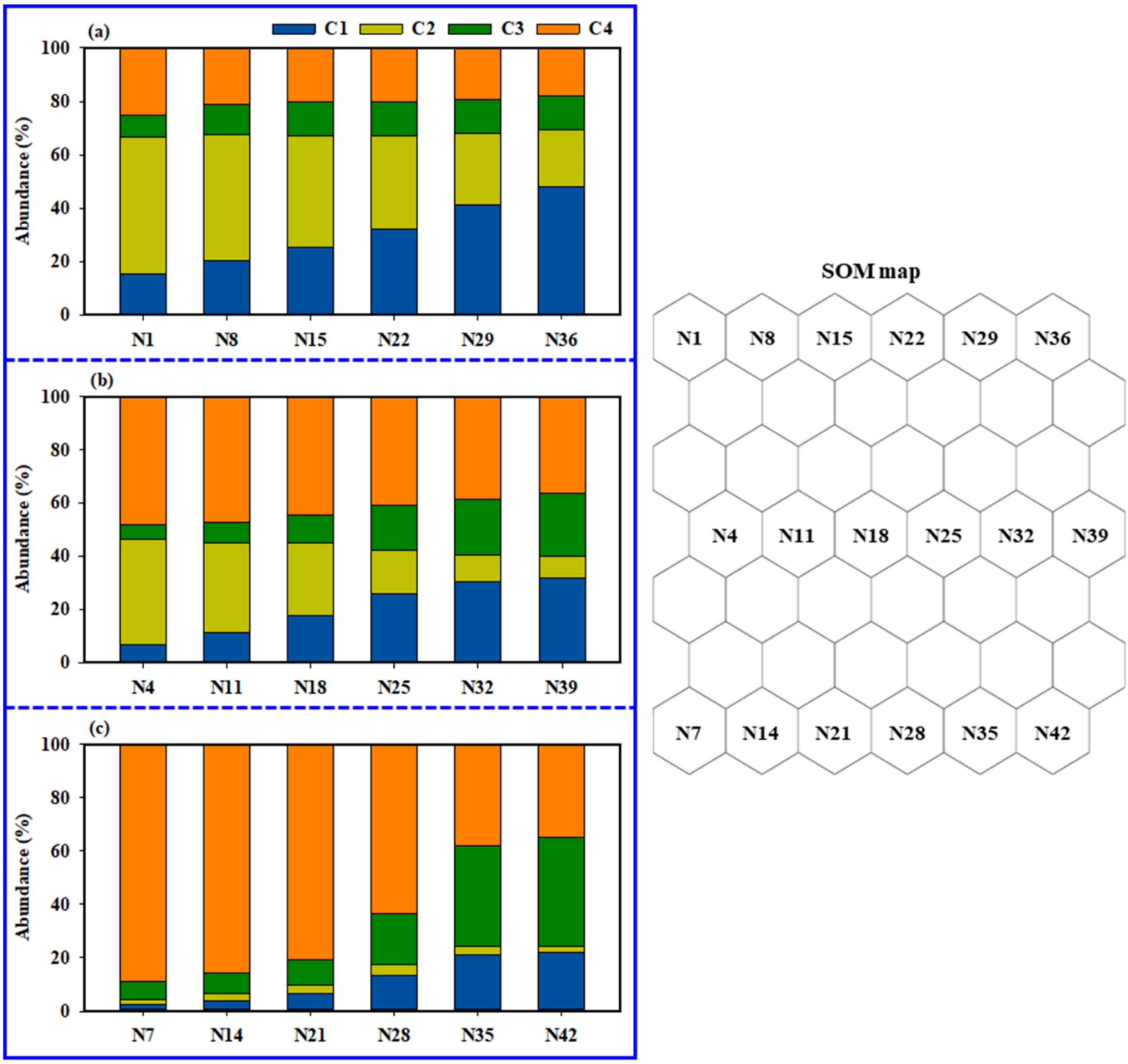

Figure S5. The abundance of reference components in typical neurons.

References

1. Huang, M.; Li, Z.; Luo, N.; Yang, R.; Wen, J.; Huang, B.; Zeng, G. Application potential of biochar in environment: Insight from degradation of biochar-derived DOM and complexation of DOM with heavy metals. Sci. Total Environ. 2019, 646, 220-228.
2. Yuan, D.; Guo, X.; Wen, L.; He, L.; Wang, J.; Li, J. Detection of Copper (II) and Cadmium (II) binding to dissolved organic matter from macrophyte decomposition by fluorescence excitation-emission matrix spectra combined with parallel factor analysis. Environ. Pollut. 2015, 204, 152-160.
3. Huang, M.; Li, Z.; Huang, B.; Luo, N.; Zhang, Q.; Zhai, X.; Zeng, G. Investigating binding characteristics of cadmium and copper to DOM derived from compost and rice straw using EEM-PARAFAC combined with two-dimensional FTIR correlation analyses. J. Hazard. Mater. 2018, 344, 539-548.
4. Quan, G.; Fan, Q.; Zimmerman, A. R.; Sun, J.; Cui, L.; Wang, H.; Gao, B.; Yan, J. 2020. Effects of laboratory biotic aging on the characteristics of biochar and its water-soluble organic products. J. Hazard. Mater. 2020, 121071.
5. Stedmon, C.A.; Bro. R. Characterizing dissolved organic matter fluorescence with parallel factor analysis: a tutorial. Limnol. Oceanogr-Meth. 2008, 6, 572-579.
6. Mahamuni, G.; Rutherford, J.; Davis, J.; Molnar, E.; Posner, J. D.; Seto, E.; Korshin, G.; Novosselov, I. Excitation-emission matrix spectroscopy for analysis of chemical composition of combustion generated particulate matter. Environ. Sci. Technol. 2020, 54, 8198-8209.
7. Murphy, K.R.; Stedmon, C.A.; Graeber, D.; Bro, R. Fluorescence spectroscopy and multi-way techniques. PARAFAC. Anal. Methods. 2013, 5, 6557.
8. He, Q.; Ding, L.; Gong, Y.; Li, W.; Wei, J.; Yu, G. Effect of torrefaction on pinewood pyrolysis kinetics and thermal behavior using thermogravimetric analysis. Bioresour. Technol. 2019, 280, 104–111.

9. Zhong, W.; Zhang, Z.; Luo, Y.; Sun, S.; Qiao, W.; Xiao, M. Effect of biological pretreatments in enhancing corn straw biogas production. Bioresour. Technol. 2011, 102, 11177–11182.
